# A systematic review and meta-analysis on the efficacy outcomes of selective serotonin reuptake inhibitors in depression in Alzheimer’s disease

**DOI:** 10.1186/s12883-023-03191-w

**Published:** 2023-05-31

**Authors:** Jinli Zhang, Xiaohui Zheng, Zhenying Zhao

**Affiliations:** 1 Department of Pharmacy, Tianjin Second People’s Hospital, Tianjin, China; 2 Department of Pharmacy, Tianjin Union Medical Center, Tianjin, China

**Keywords:** Alzheimer’s disease, SSRI, Depression, Meta-analysis

## Abstract

**Background:**

Depressive symptoms are the most common neuropsychiatric symptoms in patients with Alzheimer’s disease (AD). However, despite being common, no definite consensus recommendations exist for the management of depression in AD.

**Objective:**

To assess the effects of selective serotonin reuptake inhibitors (SSRIs) on the alleviation of depressive symptoms in patients with AD.

**Material and methods:**

Medline, Scopus, Web of Science, Google Scholar, and PsychINFO were electronically searched from inception until October 2022. Response to therapy and mean depression scores between the treatment (or before) and placebo (or after) groups were the primary outcomes. For depression scores, the standard mean deviation and accompanying 95% confidence interval were determined. The risk of bias was determined using the funnel plot, trim and fill, Egger’s and Begg’s analyses.

**Results:**

SSRIs attenuated depressive symptoms in patients with AD (0.905 SMD, 95%CI, 0.689 to 1.121, *p* < 0.000). At individual SSRI level, escitalopram, paroxetine, and sertraline significantly alleviated depressive symptoms in AD patients (0.813 SMD, 95%CI, 0.207 to 1.419, *p* = 0.009, 1.244 SMD, 95%CI, 0.939 to 1.548, *p* < 0.000, and 0.818 SMD, 95%CI, 0.274 to 1.362, *p* < 0.000). The funnel plot, trim and fill, Begg’s test (*p* = 0.052), and Egger’s test (*p* = 0.148), showed no significant risk of publication bias.

**Conclusion:**

Our meta-analysis supports the use of SSRIs for the alleviation of depression in patients with AD. However, we recommend larger randomized clinical trials that would compare the efficacy of different SSRIs in AD patients with depression.

## Key message

This study included 16 comparisons of SSRIs with placebo on 510 AD patients and showed that SSRIs attenuated depressive symptoms in these patients. However, we cannot rule out that various SSRIs within the family may have different impacts on AD.

## Introduction

Alzheimer’s disease (AD) is the most common cause of dementia worldwide. By 2050, the number of Americans who suffer from this condition is projected to reach approximately 16 million, up from the current estimate of 5.6 million [1]. Typical amnestic presentations of AD are characterized by recent memory loss, disorientation, and recall problems as its principal cognitive symptoms [2].

Nearly all individuals with AD (97%) experience neuropsychiatric symptoms (NPS). In addition, depressive and apathetic symptoms are the most common NPS in patients with AD and are present in 50% and 65% of these patients, respectively [2]. These symptoms are associated with decreased quality of life, deterioration in regular living activities, earlier institutionalization, higher mortality, and faster disease progression in patients with AD [3–5].

Currently, no definite consensus recommendations exist for the management of depression in AD patients. However, there are several pharmacological and nonpharmacological therapies for this purpose [1]. Antidepressants are routinely recommended to dementia sufferers to address their depression. Selective serotonin reuptake inhibitors (SSRIs) are suggested as the primary pharmacological therapy of choice for depression in dementia in the Work Group on Alzheimer’s Disease and Other Dementias’ 2007 practice recommendations [6]. Because they have fewer major side effects than other antidepressants, SSRIs are typically more well-tolerated [6]. Nevertheless, the National Institute for Health and Care Excellence (NICE) guideline published in June 2018 states that unless there is a pre-existing severe mental health problem, it is not recommended to regularly administer antidepressants for the management of mild to moderate depression in individuals with mild to moderate dementia [7]. Also, there is still a lack of consensus on the evidence supporting these drugs’ effectiveness. In a 12-week double-blind, placebo-controlled study of sertraline, Lyketsos et al. assessed depression symptoms using the Cornell Scale for Depression in Dementia (CSDD). The results were positive and showed that sertraline was superior to placebo [8]. On the other hand, sertraline’s potential involvement in treating depression in AD was further explored by the Depression in Alzheimer's Disease Study (DIADS)-2 study group, with published data gathered at 12 and 24 weeks. Both of them failed to show that sertraline was better than a placebo [9, 10]. In general, there was little to no effect from antidepressant medication, according to an analysis of the results from these three trials by Dudas et al. [11]. Also, we also do not have enough data regarding the outcome of individual SSRI’s. In light of these disagreements, we performed this systematic review and meta-analysis to assess the effects of SSRIs on the alleviation of depressive symptoms in patients with AD.

## Methods

### Search strategy and selection criteria

Medline via PubMed, Scopus, Web of Science, Google Scholar, and PsychINFO were electronically searched from inception until October 2022 for the papers that assessed the effects of SSRIs on the attenuation of depressive symptoms in patients with AD. We also looked for further pertinent studies in the references of each article we obtained. Further, the references included in four related meta-analyses were searched for possible inclusion of papers in the field [12–15] The key terms were “SSRIs”, “depression” and “AD”, and the search strategy was as follows: (TITLE-ABS-KEY (selective AND serotonin AND reuptake AND inhibitor) AND TITLE-ABS-KEY (depression) AND TITLE-ABS-KEY (Alzheimer’s AND disease)).

Adults with AD diagnosed in accordance with the DSM-V diagnostic criteria or the criteria of the National Institute of Neurological and Communicative Diseases and Stroke–Alzheimer’s Disease and Related Disorders Association (NINCDS-ADRDA) with concomitant depression constituted the study population [16, 17]. Additionally, Alzheimer’s Disease Assessment Scale—cognitive subscale (ADAS-cog), the Clinical Dementia Rating scale (CDR) global score [18, 19], and Mini-Mental Status Examination (MMSE) score were used to diagnose AD. The DSM-V criteria for major depressive episode (MDD), mild depression, and dysthymic disorder were all considered to be valid indicators of depression [16]. Other confirmed rating scales for depression i.e., CSDD, Hamilton depression rating scale (HDRS), Montgomery-Asberg depression rating scale (MADRS), or geriatric depression scale (GDS) were also considered valid for the definition of depression [12]. Response for treatment was defined as No response’, ‘Partial response’ (score reduction ≥ 25%), and ‘Full response’ (score reduction ≥ 50%) in CSDD [18, 20], mild depression (17–24), moderate depression (25–30), and severe depression (≥ 31) in HDRS [21], or ≥ 50% reduction in HAM-D score [22]. For the other scales, no clear definition of response-to-treatment was found. Only full-text English studies on humans were included in this meta-analysis. In vivo, in vitro, and in silico studies were excluded and not considered for further evaluation. Our analysis did not include agents that could not be classified as SSRIs. The study was in accordance with the Preferred Reporting Items for Systematic Reviews and Meta-Analyses (PRISMA) recommendations [23].

### Data extraction

Abstracts were initially scrutinized for specific inclusion criteria. To attain high quality and minimal heterogeneity, papers were then evaluated based on the previously indicated exclusion criteria. The kappa measure of agreement was determined to guarantee consistency in coding after two authors independently coded the studies (κ = 1.00 [100% consistency]). Additionally, the studies’ authors were contacted for raw or continuous data where data were not available [10] Data such as baseline participant characteristics, study drug, duration of treatment, and depression scores were retrieved from each report. To calculate the effect size, we gathered sample numbers, means, and standard deviations (SDs) for each treatment group. Other statistical information that might be transformed into means and SDs was retrieved if these values were absent. Studies were disqualified if the authors could not compute, impute, or provide the missing outcome data.

### Outcomes

Response to therapy and mean depression scores between the treatment (or before) and placebo (or after) groups were the primary outcomes.

### Risk of bias assessment

Two authors independently evaluated the included studies’ risk of bias (RoB) using the Cochrane RoB assessment technique (sequence generation, allocation concealment, blinding, selective reporting bias, and attrition bias). We looked for asymmetry in funnel plots to visually assess the possibility of publication bias. Also, trim and fill analysis was used to assess publication bias in the included studies.

### Data analysis

Effect size (ES) estimations and publication bias testing were performed using Comprehensive Meta-Analysis (CMA) (Version 2; Biostat Inc., Englewood, NJ, USA). For depression scores, the standard mean deviation (SMD) and accompanying 95% confidence interval (CI) were determined [24] In our meta-analytic systematic review, a random-effect model that accepts population-level inferences and is more strict than a fixed effect model was employed to account for heterogeneity in ES estimations [25]. The *I*^2^ statistic was used to quantify heterogeneity in the degree of inconsistency (total variation across studies that is due to heterogeneity rather than chance), which suggests rejection of the homogeneity hypothesis of the effect set in the presence of a significant Q-test value. This statistic's values fall between 0 and 100%, with low, moderate, and high *I*^2^ levels of 25, 50, and 75%, respectively [25]. In the absence of the mean and SD, data from the median and interquartile ranges were translated to the mean and SD based on distributional assumptions. The following calculations were performed for several outcomes given for a single purpose in the same study:

1 $$\overline{Y}=\frac{1}{m}\left(\sum\nolimits_{j}^{m}{Y}_{j}\right)$$

where "m" is the number of means and "Y" is the average of the effect sizes from the various outcomes. The entire variance of these means was nonetheless determined as follows:

2 $$V_{\overline{Y}}=\left(\frac{1}{m}\right)^{2}var\left({\textstyle\sum_{j=1}^m}Y_i\right)=\left(\frac{1}{m}\right)^2\left({\textstyle\sum_{j=1}^m}V_i+{\textstyle\sum_{j\neq k}}\left(r_{jk}\sqrt{V_j}\sqrt{V_k}\right)\right)$$

where "m" is the number of variations in the formula and "V" denotes a variation. In each study, *p* < 0.05 was considered statistically significant.

## Results

**Fig. 1:**
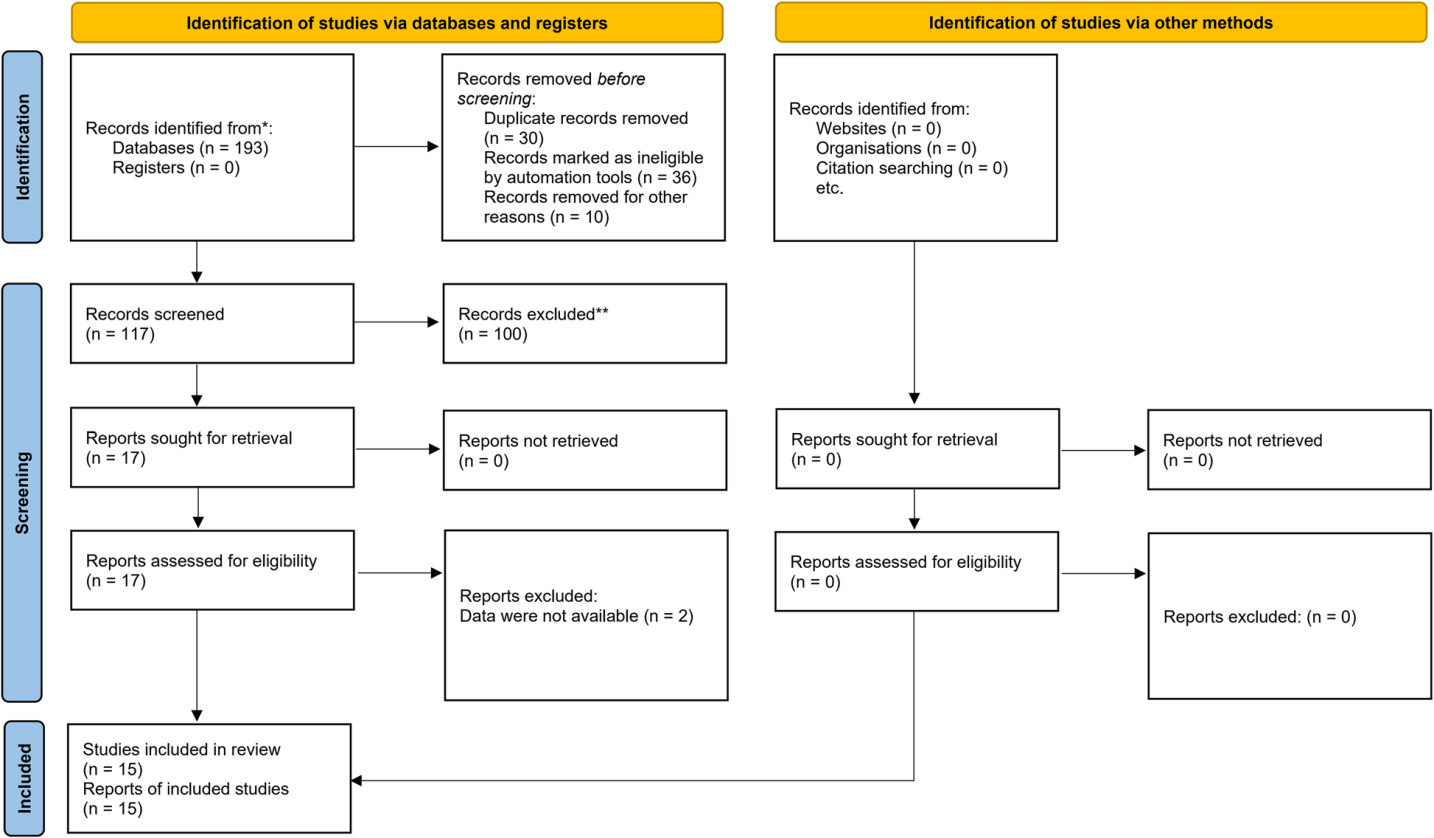
Selection process of the included articles in the systematic review and meta-analysis

A total of 193 records were found throughout the search, and after the first screening of titles and abstracts, 117 publications were thought to be possibly relevant. Accordingly, 100 studies were excluded for several reasons. Some studies were not relevant or they were duplicates. Also, there were some publications that were not original contributions and were reviews. Some other articles had used other antidepressants to treat depression in AD. Others tried SSRIs in disorders other than AD. Thus, 17 studies were included in the meta-analysis. However, two studies did not report the required data for meta-analysis and were excluded [10, 26] This meta-analysis is comprised of 15 publications and 16 distinct treatments to examine how SSRIs affect depressive symptoms in AD patients. Figure 1 shows the PRISMA flowchart for the research inclusion process. Table 1 presents the study characteristics in the included publications. In this meta-analysis, 510 patients with mild to moderate AD and depression were examined. Fourteen studies were randomized clinical trials (RCTs) [2, 9, 18–22, 27–33] One study was prospective cohort with before/after design [34] 4, 1, 3, 1, and 7 trials utilized escitalopram, citalopram, fluoxetine, paroxetine, and sertraline, respectively. In the majority of the included trials, SSRI therapy alleviated depression symptoms.

**Table 1:** Summary of demographic data and findings of the included studies in the meta-analysis

Study	Study design	Age (year)	No. of participants		Treatment	Dose (mg)	Duration (weeks)	Depression scale	AD duration (year)	Treatment effect
			Placebo/before	Treatment/after						
An et al., 2017 [18]	RCT	74.33 ± 7.47	33	27	Escitalopram	5–15	12	CSDD	-	No effect
Banerjee et al., 2011 [27]	RCT	80 ± 8.4	111	107	Sertraline	50–150	13	CSDD	-	No effect
Choe et al., 2016 [19]	RCT	74.33 ± 7.12	29	28	Escitalopram	20	52	CSDD	-	+
Katona et al., 1998 [28]	RCT	76.6	99	99	Paroxetine	20–40	8	MADRS	-	+
Lyketsos et al., 2003 [20]	RCT	75.5 ± 9.5	20	24	Sertraline	95	12	CSDD, HDRS	34.6 ± 21.4	+
Magai et al., 2000 [29]	RCT	88.4 ± 6.1	14	17	Sertraline	25–100	8	CSDD, GS	-	No effect
Mokhber et al., 2014 [21]	RCT	67.3 ± 3.0	20	20	Sertraline	150	12	HDRS	-	+
Mowla et al., 2007 [30]	RCT	69.2	41	40	Fluoxetine	20	12	HDRS	-	No effect
Munro et al., 2004 [31]	RCT	75.5 ± 9.7	18	23	Sertraline	25–150	12	CSDD, HDRS	-	+
Nyth and Gottfries, 1990 [32]	RCT	77.2	29	26	Citalopram	20	16	MADRS	-	+
Petracca et al., 2001 [22]	RCT	70.2 ± 6.3	24	17	Fluoxetine	10–40	6	HDRS	-	No effect
Rao et al., 2006 [34]	Before/After	50–90	15	15	Escitalopram	10–20	8	CSDD	-	+
Rosenberg et al., 2010 [9]	RCT	77.3 ± 8.0	64	67	Sertraline	50–100	12	CSDD	-	No effect
Takemoto et al., 2020 a [2]	RCT with before/after design	73.0 ± 7.3	11	11	Sertraline	31.8	12	HDRS	-	No effect
Takemoto et al., 2020 b [2]	RCT with before/after design	79.1 ± 6.1	13	13	Escitalopram	7.3	12	GDS	-	+
Taragano et al., 1997 [33]	RCT	71.7 ± 5.0	18	18	Fluoxetine	10	6	HDRS	-	+

The data are expressed as mean ± standard deviation (SD)

*RCT* Randomized clinical trial, *AD* Alzheimer’s disease, *HAM-D* Hamilton depression rating scale (HDRS), *MADRS* Montgomery-Asberg depression rating scale, *GDS* Global depression scale, *CSDD* Cornell Scale for Depression in Dementia, *No.* Number

### SSRIs efficacy

**Fig. 2:**
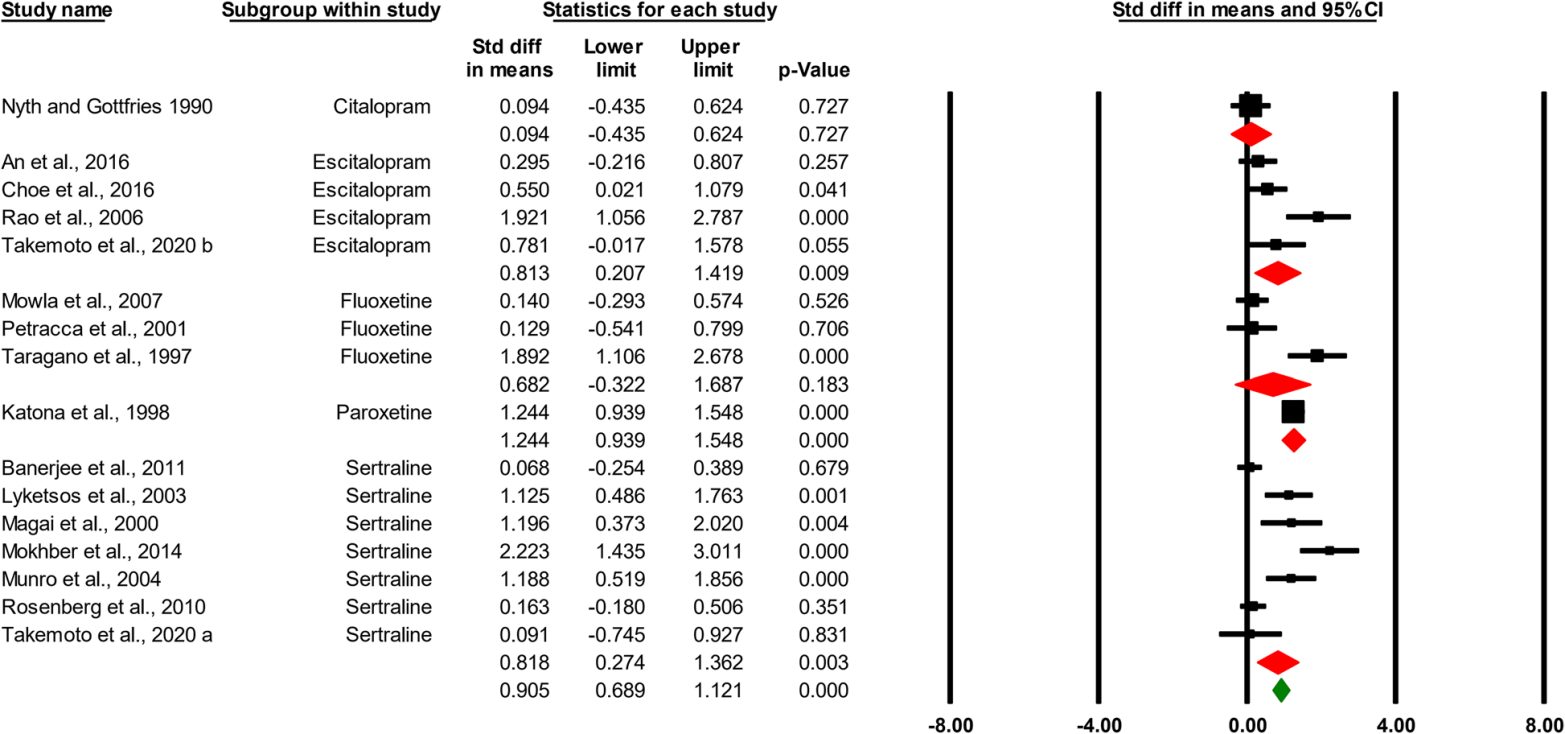
The effect size for selective serotonin reuptake inhibitors impacts on depressive symptoms in AD patients shown by a forest plot of the standardized mean difference (SMD). The red squares represent the pooled effects for each research subgroup, whereas the green square represents the overall pooled impact. Each study’s SMD is shown by a black square. 95% confidence intervals are represented by horizontal lines (CI)

All depression measures were included in the initial global nested analysis using CMA, which counted each study once. We found that SSRIs treatment attenuated depressive symptoms in patients with AD and concomitant depression (0.905 SMD, 95%CI, 0.689 to 1.121, *p* < 0.000) (Fig. 2). As expected, results emerging from heterogeneity analysis showed a high degree of heterogeneity among the included studies (ϰ^2^ = 89.807, T^2^ = 0.354, d.f. = 15, *I*^*2*^ = 83.29%, *p* < 0.000).

We also performed the analysis at the individual SSRI level. We found that citalopram treatment was not effective in reducing depressive symptoms in patients with AD (0.094 SMD, 95%CI, -0.435 to 0.624, *p* = 0.727). This was the same for fluoxetine treatment (0.682 SMD, 95%CI, -0.322 to 1.687, *p* = 0.183). However, escitalopram, paroxetine, and sertraline significantly alleviated depressive symptoms in AD patients (0.813 SMD, 95%CI, 0.207 to 1.419, *p* = 0.009, 1.244 SMD, 95%CI, 0.939 to 1.548, *p* < 0.000, and 0.818 SMD, 95%CI, 0.274 to 1.362, *p* < 0.000) (Fig. 2).

### Leave-one-out sensitivity analysis

**Fig. 3:**
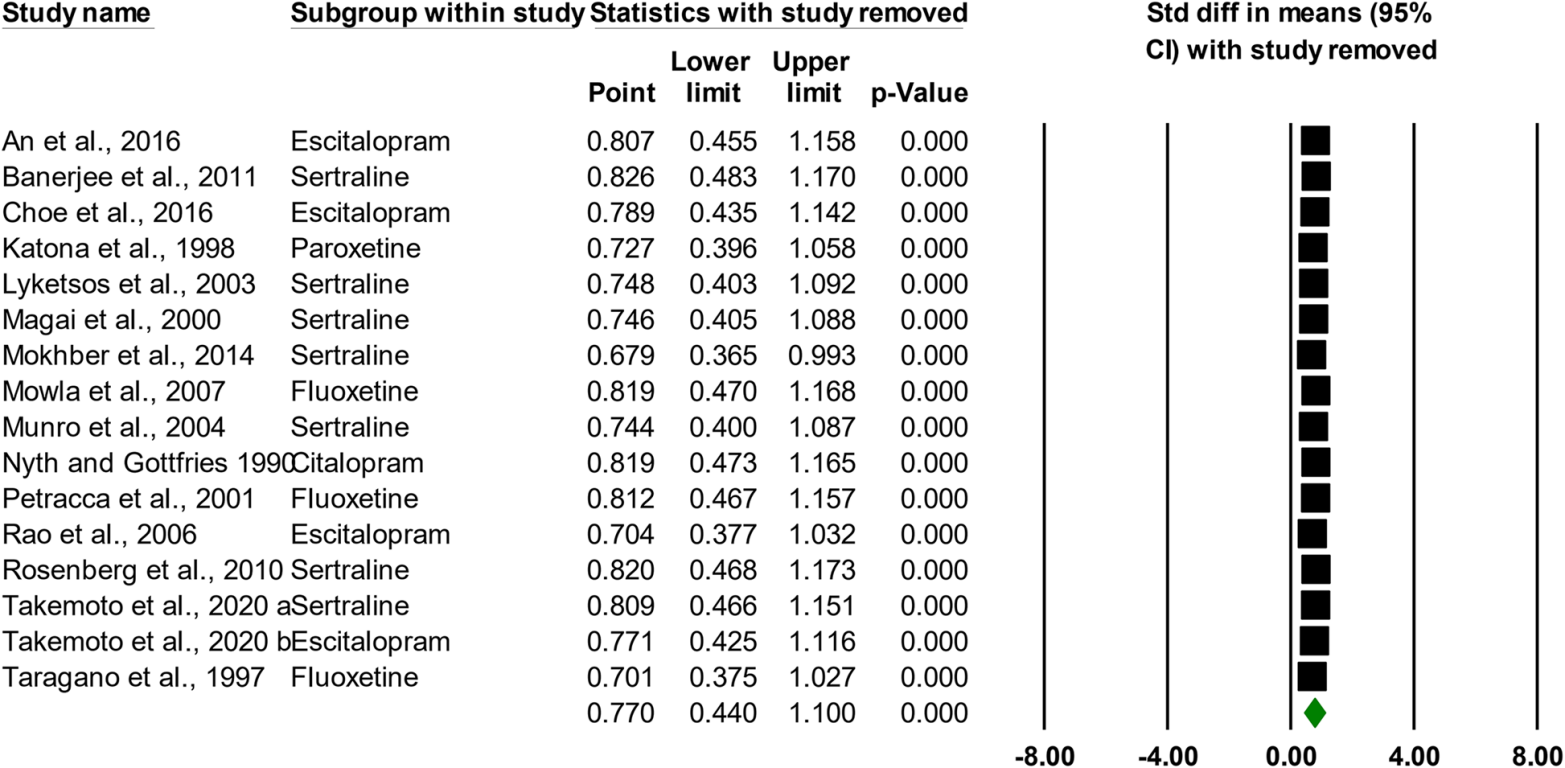
The “comprehensive meta-analysis (CMA)” software’s “leave-one-out” sensitivity analysis “one study deleted” feature

Analysis that excluded each trial showed that no one study was responsible for these findings, which instead represented a pattern of overall combined, opposing associations between the use of SSRIs and the intensity of depressive symptoms in AD patients (Fig. 3).

### Publication bias

**Fig. 4:**
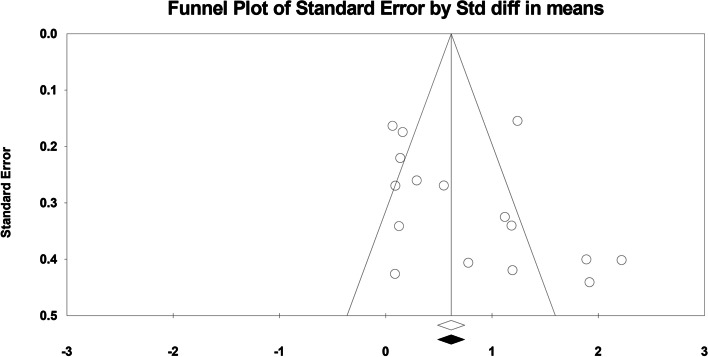
Funnel Plot of standard error against SMD after Duval and Tweedie’s trim and fill. SMD, standard difference of mean

**Fig. 5:**
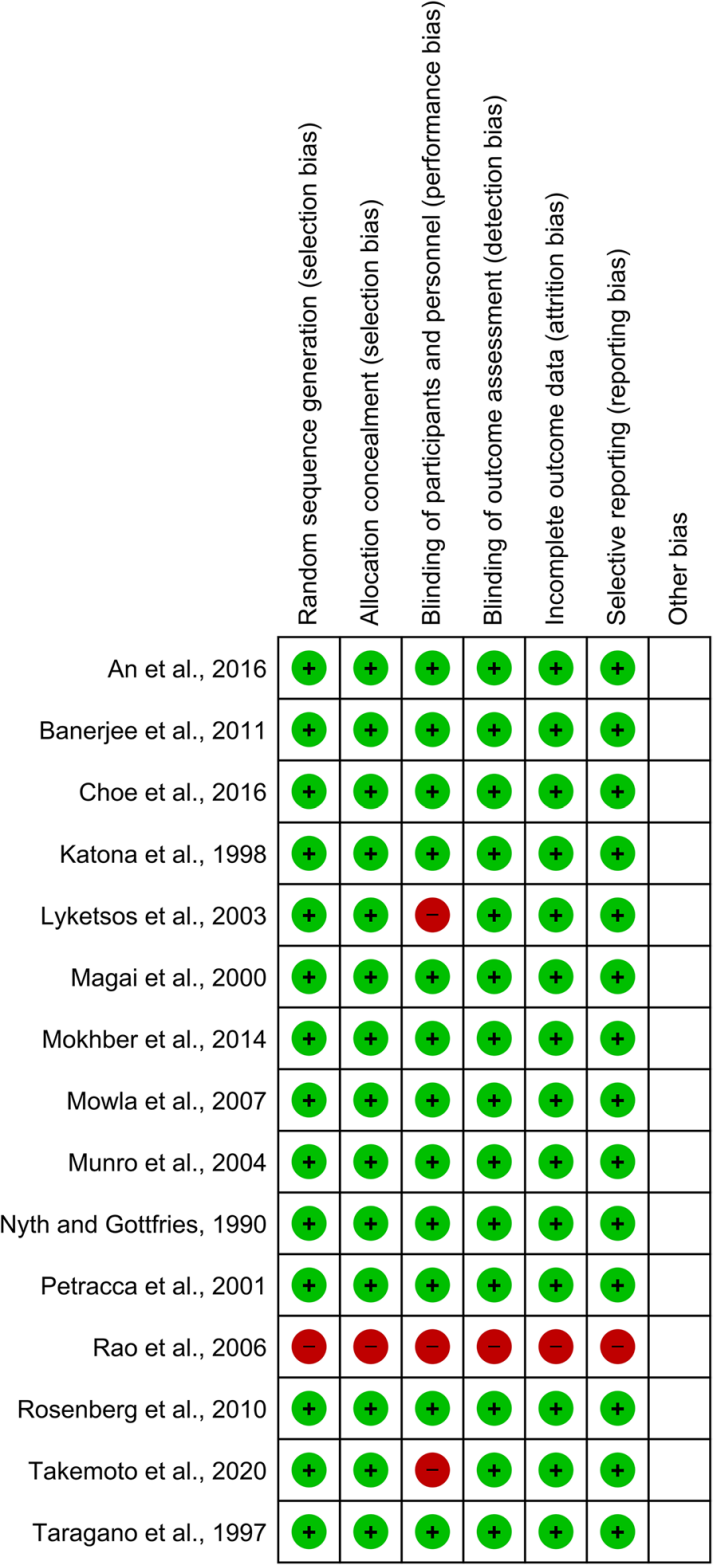
Different levels of bias risk for each factor in the studies that were considered. The Cochrane risk of bias tool was used in order to determine publication bias

The Cochrane RoB Tool revealed that the overall quality of the articles was good. Only one study had a high level of publication bias [34] Apart from this study, two other publications did not observe blinding of participants and personnel (performance bias) [2, 20] The lack of the putative pleiotropic effects was also shown by the funnel plot, which revealed no indication of noticeable heterogeneity between the estimations (Figs. 4 and 5). In the trim and fill analysis, no study was trimmed to any side of the mean. Begg’s test (*p* = 0.052) and Egger’s test (*p* = 0.148), showed no significant risk of publication bias.

## Discussion

### Main findings and their interpretation

We assessed the existing evidence on SSRI monotherapy for the treatment of depressive symptoms in AD patients with concomitant depression using a thorough meta-analytic methodology. This study only included patients with probable mild to moderate AD and it did not cases with mild cognitive impairment or severe AD. So, the results might not be generalizable to these populations. We found that SSRI treatment significantly attenuated depressive symptoms in patients with AD and concurrent depression, bearing in mind that the full body of evidence emerging from these studies had a high level of heterogeneity.

Depression is frequently observed in AD and has a considerable negative impact on morbidity and mortality [14] In that light, SSRIs are suggested as the primary pharmacological therapy of choice for depression in dementia [6]. SSRIs block serotonin reuptake from synapses by targeting the serotonin transporters. Potential side effects of these medications can include gastrointestinal issues such as nausea, vomiting, stomach discomfort, and diarrhea, as well as migraines, anxiety, dry mouth, appetite loss, and dizziness, all of which are often moderate [1]. Additionally, SSRIs are less likely to result in disorientation or falling since they have less significant anticholinergic and antiadrenergic characteristics [1]. Further, it has been well shown that SSRI therapy combined with acetylcholinesterase inhibitors has beneficial benefits on cognition in AD patients [35, 36]. However, it should be noted that many patients (with or without dementia) achieve only a partial response or perhaps no response at all with SSRIs. Thus, it is quite trendy to add a 2nd class of drug or a low-dose antipsychotic such as aripiprazole, to augment antidepressant treatment [37].

SSRIs (with or without other antidepressants) have been the subject of prior meta-analytic investigations that evaluated their effectiveness and safety in treating depression in AD [12–15]. Sepehry et al., stated that the effectiveness of SSRI therapy for treating comorbid depressive symptoms in AD is not yet supported by evidence. Nevertheless, it should be considered that even within the same class of drug, there is a significant difference in each person's clinical reaction and tolerance [15]. Sepehry et al. argued that this could be because there isn’t agreement on the diagnostic strategy and results of antidepressant studies in AD. In that light, results will be more trustworthy when there is a standardized diagnostic methodology [30]. However, one limitation of this study was that the analyses for depression in this study contained a small number of publications, and the analyses were conducted separately for HDRS and CSDD [15]. In another meta-analysis, Orgeta et al. concluded lack of any conclusive proof that antidepressants are effective at treating depression in AD [13]. This analysis, however, was performed on a limited number of studies and its results were based on a heterogeneous group of antidepressants. Contrary to the results of previous meta-analyses, He et al., suggested that sertraline ought to be used as an alternate therapy for depression in patients with AD [12] Similarly, Thompson et al., found that compared to placebo treatment, antidepressant therapy was effective for AD [14]. The evidence from these studies was also mixed, as it included antidepressants other than SSRIs in its analyses.

It is worth to note that antidepressants are often administered despite the scant and conflicting data on their effectiveness in AD. According to David et al., prescription rates increased significantly from 26% in 2010 to 31% in 2014 [38]. This was confirmed in other studies [39, 40].

### Strengths and limitations

Because we included the largest and most-recent clinical studies on the subject that has been published to date, our analyses’ external validity is stronger than that of earlier research in the field. The evidence supporting the use of SSRIs as a class of drugs for the treatment of AD with concomitant depression appears to be relatively robust in light of our findings. The fact that we focused solely on SSRIs and dementia caused by AD with a diagnosis of concomitant depression is a key strength of our study since it allowed us to assess the therapy of depression in a clearly defined clinical entity. Depression is likely experienced differently across the continuum of cognitive decline from mild cognitive impairment to AD and in different dementias [41]. This study’s superior methodological quality, achieved by adhering to the PRISMA consensus declaration, is one of its other strong points.

There are a few limitations, though. First, because each SSRI molecule has varied pharmacological characteristics, we cannot completely rule out that various SSRI compounds within the family may have different impacts on AD. As we showed, some SSRIs such as citalopram and fluoxetine do not have significant effects in this regard, whereas others such as sertraline and escitalopram do show this impact. Only a small number of trials have explored different types of SSRIs; the majority of them have focused on sertraline [2]. Second, there is no gold standard for diagnosing depression in AD. These analyses’ research used DSM-IV criteria which need fewer symptoms to diagnose depression [42]. CSDD, HDRS (HAM-D), MADRS, and GDS have been used in the investigations that constitute this study. The possibility that the effect of SSRI medication is scale-dependent is out of the question, and our data provide some evidence for it. The HDRS focuses a lot of emphasis on neurovegetative symptoms, and it’s feasible that treating with SSRIs will help these rather than mood issues [15]. However, without patient-level information on particular neurovegetative symptoms, we are unable to further evaluate this hypothesis. Also, we did not look at moderating variables due to statistical limitations, but we cannot rule out the potential that how depression is identified in AD may have an impact on the results. Another limitation of this study was that an outdated definition of Alzheimer’s disease that is devoid of biomarker data (i.e., the NINCDS criteria) was used as a diagnosis tool for AD. However, this was unavoidable given the publication time of many of the included studies. It was also impossible for us to know what cognitive syndrome those with AD had, though we would presume much of the literature was about those with dementia thought to be due to AD. And last but not least it is difficult to make a conclusion on the efficacy of citalopram and fluoxetine with only one study included for each medication in this analysis.

## Conclusion

Evidence emerging from our meta-analysis supports the use of SSRIs for the alleviation of depression in patients with AD. Considering their favorable safety profiles over other classes of antidepressants and their positive effects on cognition when combined with acetylcholine esterase medications, SSRI use is recommended in AD patients with concomitant depression. However, even within the same class of drugs, there is a significant difference in how each person responds clinically and tolerates a given prescription. So, the necessity for conducting larger RCTs that compare the efficacy of different SSRIs in AD patients with depression is felt. Also, it should be noted that even with SSRIs treatment, additional augmentation with other agents may have to be considered in some of the patients.

## Data Availability

The public datasets used in this study are available. The other data supporting this study could be requested from the corresponding author.
